# Post‐Disclosure Distress in Family Members of Individuals Receiving Amyloid PET Scans for Cognitive Changes

**DOI:** 10.1002/alz70858_105640

**Published:** 2025-12-26

**Authors:** Cristina de Rosa, Dianxu Ren, Josh D Grill, Jennifer H Lingler

**Affiliations:** ^1^ University of Pittsburgh, Pittsburgh, PA, USA; ^2^ University of California, Irvine, Irvine, CA, USA

## Abstract

**Background:**

Biomarker testing like amyloid imaging is increasingly common in clinical evaluations of people with cognition and memory changes. Family members, including kin‐like friends, of individuals being evaluated may be affected by this process, as scan results may have implications for managing current situational demands and anticipating caregiving responsibilities. However, very little is known about how amyloid PET results disclosure impacts family members, particularly regarding emotional distress. This study aimed to identify correlates of post‐disclosure distress in family members of individuals undergoing amyloid imaging as part of cognitive symptom evaluation.

**Method:**

This analysis pooled preliminary data from a multisite study of Patient And Family Member Reactions to Biomarker‐informed ADRD DiagnosEs (PARADE) and data from the completed Return of Amyloid Imaging Scan Results (RAISR) study. Family members of individuals receiving amyloid scan results were considered eligible for this sample if they completed data collection interviews before and after result disclosure (*n* = 57). Hierarchical linear regression models tested associations between family members’ self‐reported age, gender, race/ethnicity, understanding of the scan result as amyloid elevated or not, anxiety (State‐Trait Anxiety Inventory‐Short), and depression (Center for Epidemiological Studies‐Depression) in block 1, and coping styles (Brief COPE) in block 2, with the impact of disclosure measured by the Impact of Event Scale (IES) and Impact of Genetic Testing‐AD (IGT‐AD) distress and positive subscales adapted for amyloid PET.

**Result:**

Most family members were female (66.7%) and White/non‐Hispanic (73.7%). Older age (*M* = 66.2) was associated with distress on both the IES (*b* = 0.36 [0.16, 0.56], *p* = .005) and adapted IGT‐AD (*b* = 0.25 [‐0.17, 0.45], *p* = .042, although this bootstrap confidence interval suggested some uncertainty about the observed effect). Learning a positive amyloid result (*b* = 13.55 [5.17, 24.50], *p* = .007) and having more depressive symptoms prior to result disclosure (*b* = 0.86 [0.51, 1.35], *p* < .001) also predicted greater test‐related distress. Positive scan results were additionally associated with IGT‐AD positive subscale ratings indicating more distress (*b* = 3.97 [0.99, 7.18], *p* = .017).

**Conclusion:**

Family members who are older and experiencing depressive symptoms may be at higher risk of distress following their relative's amyloid PET result disclosure, particularly when results are positive. Future work is needed to identify actionable intervention targets.

